# Global and regional right ventricular mechanics in repaired tetralogy of Fallot with chronic severe pulmonary regurgitation: a three-dimensional echocardiography study

**DOI:** 10.1186/s12947-021-00260-3

**Published:** 2021-08-06

**Authors:** Jurate Bidviene, Denisa Muraru, Attila Kovacs, Bálint Lakatos, Egle Ereminiene, Csilla Liptai, Jolanta-Justina Vaskelyte, Remigijus Zaliunas, Elena Surkova, Luigi P. Badano

**Affiliations:** 1 Department of Cardiology, Medical Academy, Lithuanian University of Health Sciences, Eiveniu str. 2, 50009 Kaunas, Lithuania; 2 Institute of Cardiology, Medical Academy, Lithuanian University of Health Sciences, Kaunas, Lithuania; 3 Department of Cardiological, Neural and Metabolic Sciences, Istituto Auxologico Italiano, IRCCS, Milan, Italy; 4 Department of Medicine and Surgery, University Milano-Bicocca, Milan, Italy; 5 Heart and Vascular Center, Semmelweis University, Budapest, Hungary; 6 Royal Brompton Hospital, Part of Guy’s and St Thomas’ NHS Foundation Trust, London, SW3 6NP UK

**Keywords:** Right ventricular mechanics, Severe pulmonary regurgitation, Tetralogy of Fallot, Right ventricular strain, 3D echocardiography

## Abstract

**Background:**

Data about the right ventricular (RV) mechanics adaptation to volume overload in patients with repaired tetralogy of Fallot (rToF) are limited. Accordingly, we sought to assess the mechanics of the functional remodeling occurring in the RV of rToF with severe pulmonary regurgitation.

**Methods:**

We used three-dimensional transthoracic echocardiography (3DTE) to obtain RV data sets from 33 rToF patients and 30 age- and sex- matched controls. A 3D mesh model of the RV was generated, and RV global and regional longitudinal (LS) and circumferential (CS) strain components, and the relative contribution of longitudinal (LEF), radial (REF) and anteroposterior (AEF) wall motion to global RV ejection fraction (RVEF) were computed using the ReVISION method.

**Results:**

Corresponding to decreased global RVEF (45 ± 6% vs 55 ± 5%, p < 0.0001), rToF patients demonstrated lower absolute values of LEF (17 ± 4 vs 28 ± 4), REF (20 ± 5 vs 25 ± 4) and AEF (17 ± 5 vs 21 ± 4) than controls (p < 0.01). However, only the relative contribution of LEF to global RVEF (0.39 ± 0.09 vs 0.52 ± 0.05, p < 0.0001) was significantly decreased in rToF, whereas the contribution of REF (0.45 ± 0.08 vs 0.46 ± 0.04, p > 0.05) and AEF (0.38 ± 0.09 vs 0.39 ± 0.04, p > 0.05) to global RVEF was similar to controls. Accordingly, rToF patients showed lower 3D RV global LS (-16.94 ± 2.9 vs -23.22 ± 2.9, p < 0.0001) and CS (-19.79 ± 3.3 vs -22.81 ± 3.5, p < 0.01) than controls. However, looking at the regional RV deformation, the 3D CS was lower in rToF than in controls only in the basal RV free-wall segment (p < 0.01). 3D RV LS was reduced in all RV free-wall segments in rToF (p < 0.0001), but similar to controls in the septum (p > 0.05).

**Conclusions:**

3DTE allows a quantitative evaluation of the mechanics of global RVEF. In rToF with chronic volume overload, the relative contribution of the longitudinal shortening to global RVEF is affected more than either the radial or the anteroposterior components.

## Background

The chronic pulmonary regurgitation and consequent right ventricular (RV) failure in patients after repair of tetralogy of Fallot is an important cause of morbidity and late mortality [1]. Pulmonary valve replacement has become the treatment of choice to treat late pulmonary regurgitation in these patients, however, there are no clear, evidence-based guidelines on the optimal timing for this procedure [2]. Thus, an accurate assessment of RV function plays an important role in selecting the most appropriate time to replace the pulmonary valve.

A previous study demonstrated that the deterioration of RV longitudinal strain has been found in repaired tetralogy of Fallot (rToF) patients with maintained RV ejection fraction (RVEF) [3], suggesting that other wall motion components help to maintain the pump function. Moreover, recently, using the three-dimensional transthoracic echocardiography (3DTE) Lakatos et al. demonstrated that the radial and anteroposterior motions directions of the RV have comparable significance to longitudinal shortening in determining global RV function in healthy volunteers [4]. These findings confirmed that standard parameters referring only to longitudinal RV shortening are not sufficient to evaluate the RV function thoroughly.

However, due to the complex RV anatomy and mechanics it is difficult to obtain a comprehensive imaging of the RV by two-dimensional transthoracic echocardiography (2DTE) [5]. Accordingly, most of the conventional echocardiographic parameters using in everyday clinical practice describe RV longitudinal function only [6] and other components of RV mechanics, such as the inward movement of the RV free wall (radial motion) and the traction of the RV free wall insertion points into the ventricular septum (anteroposterior motion) [7] are usually neglected. Fortunately, recent advancements in 3DTE allow us to encompass the entire RV (inflow, outflow, and apex) in a single pyramidal dataset and offers an alternative, more comprehensive method to quantify RV function. It is not limited to the assessment of the longitudinal shortening anymore, and provides a good correlation with RV volumes and ejection fraction measured by cardiac magnetic resonance imaging [8]. Nevertheless, little is known about how the RV mechanics (especially the radial and anteroposterior contraction) and 3D strain changes in rToF patients with RV volume overload due to postoperative pulmonary regurgitation. According to our knowledge, no 3DTE study addressed the changes in global (evaluating separately longitudinal, radial and anteroposterior contraction) and regional RV mechanics, and their relations with RV function in these patients.

We hypothesized that chronic volume overload affects the RV mechanics and that these changes are related to changes in RV function. Accordingly, our aim was to investigate the global and regional RV mechanics in rToF patients with chronic RV volume overload compared with healthy volunteers using 3D echocardiography.

## Methods

### Study population

We performed a prospective longitudinal study on rToF patients followed up at our centre (University Hospital of Padua, Italy) between May 2016 and January 2017. We obtained 3DTE data sets of the RV from 33 rToF patients (58% women, age 20 ± 8 years) with chronic severe pulmonary regurgitation without significantly elevated RV systolic pressure [9]. In 29 (88%) rToF patients surgery was performed using transarterial approach, whereas the transventricular approach was used in the remaining 4 patients (12%) (surgery age was 8 ± 11 months, duration from the surgery to the time of study recruitment 18.6 ± 7.3 years). Transannular patches were placed in 25 (76%) of patients, whereas infundibulectomy was used in 4 (12%) patients (in all the latter patients the native pulmonary valve was preserved and none of them underwent valvulotomy). The surgical technique applied to the remaining 4 (12%) patients could not be adequately categorized from previous medical records. All rToF patients had severe pulmonary regurgitation, 13 (39%) patients had moderate and 1 (3%) had severe tricuspid regurgitation.

Patients with either pulmonary stenosis or prosthetic pulmonary valve, greater than mild aortic or mitral valve disease, residual intracardiac shunts, atrial fibrillation, left ventricular ejection fraction < 50%, pacemaker, or poor quality of echocardiographic images were excluded from the study.

The control group consisted of 30 age- and sex- matched healthy volunteers (63% women, age 21 ± 6 years) selected from the Padua “3D Echo Normal” database [10]. Recruited subjects were asymptomatic (with no prior history of cardiovascular or lung disease), had normal physical examination, electrocardiogram and transthoracic echocardiography.

Our study protocol was approved by the University of Padua Ethics Committee (protocol 2380P approved on 06/10/2011). Written informed consent was obtained from all volunteers.

### Echocardiography

Transthoracic echocardiography examinations were performed by an experienced sonographer using the Vivid E9 scanner (GE Vingmed, Horten, Norway) equipped with M5S-D and 4 V-D probes with patients in the left lateral decubitus position. In addition to a complete standard 2D and Doppler examination, dedicated 2D and 3DTE acquisitions were obtained according to the study protocol. Digital loops were stored in a dedicated workstation and analyzed offline.

To assess the RV systolic function, fractional area change (FAC), M-mode-derived tricuspid annular plane systolic excursion (TAPSE) and systolic pulsed wave tissue Doppler velocity of the lateral tricuspid annulus (s′) were measured [6].

Three consecutive cardiac cycles, ensuring a frame rate between 50 and 80 Hz, were recorded in dedicated apical 4-chamber RV-focused view for RV longitudinal strain (LS) quantifications. Assessment of RV global LS, RV interventricular septum and free wall LS was performed by 2D speckle tracking echocardiography using Q-Analysis package (EchoPAC BT13; GE Vingmed) according to recently published recommendations [11]. Subjects with inadequate tracking of > 2 segments per region of interest were excluded from analysis.

Tricuspid and pulmonary regurgitation were quantified using color Doppler and continuous wave Doppler traces according to current guidelines [12, 13]. Pulmonary valve regurgitation was defined as severe when met these criteria: large, with a wide origin colour flow pulmonary regurgitation jet (a jet width that occupies > 65% of the RV outflow tract width measured in the same frame); dense continuous-wave Doppler signal /steep deceleration, early termination of diastolic flow; and, origin of the reverse diastolic flow in the branches of the pulmonary artery as assessed by color Doppler flow imaging. RV systolic pressure was calculated from the peak velocity of tricuspid regurgitation signal and right atrial pressure, estimated from the dimensions and respiratory changes of inferior vena cava [6].

Beyond the conventional echocardiographic protocol, multi-beat (4 or 6) full-volume RV and left ventricular data sets were acquired during end-expiratory breath-hold from the apical approach, including the entire structure in the acquisition, with particular attention to RV anterior wall and outflow tract. Each acquisition was verified to rule out stitching artifacts or incomplete RV visualization. Image depth and sector were optimized to achieve frame rates ≥ 20 Hz.

Offline analysis of 3D RV end-systolic and end-diastolic volumes, and ejection fraction were performed by a single experienced investigator using a commercially available 3DE software package (TomTec 4D RV-Function 2, Unterschleissheim, Germany) [14]. Quantification of 3D left ventricular volumes and ejection fraction was performed using 4D Auto LVQ (GE Healthcare) [15]. All volumes were indexed for body surface area.

### Evaluation of 3D RV mechanics and strain

**Fig. 1:**
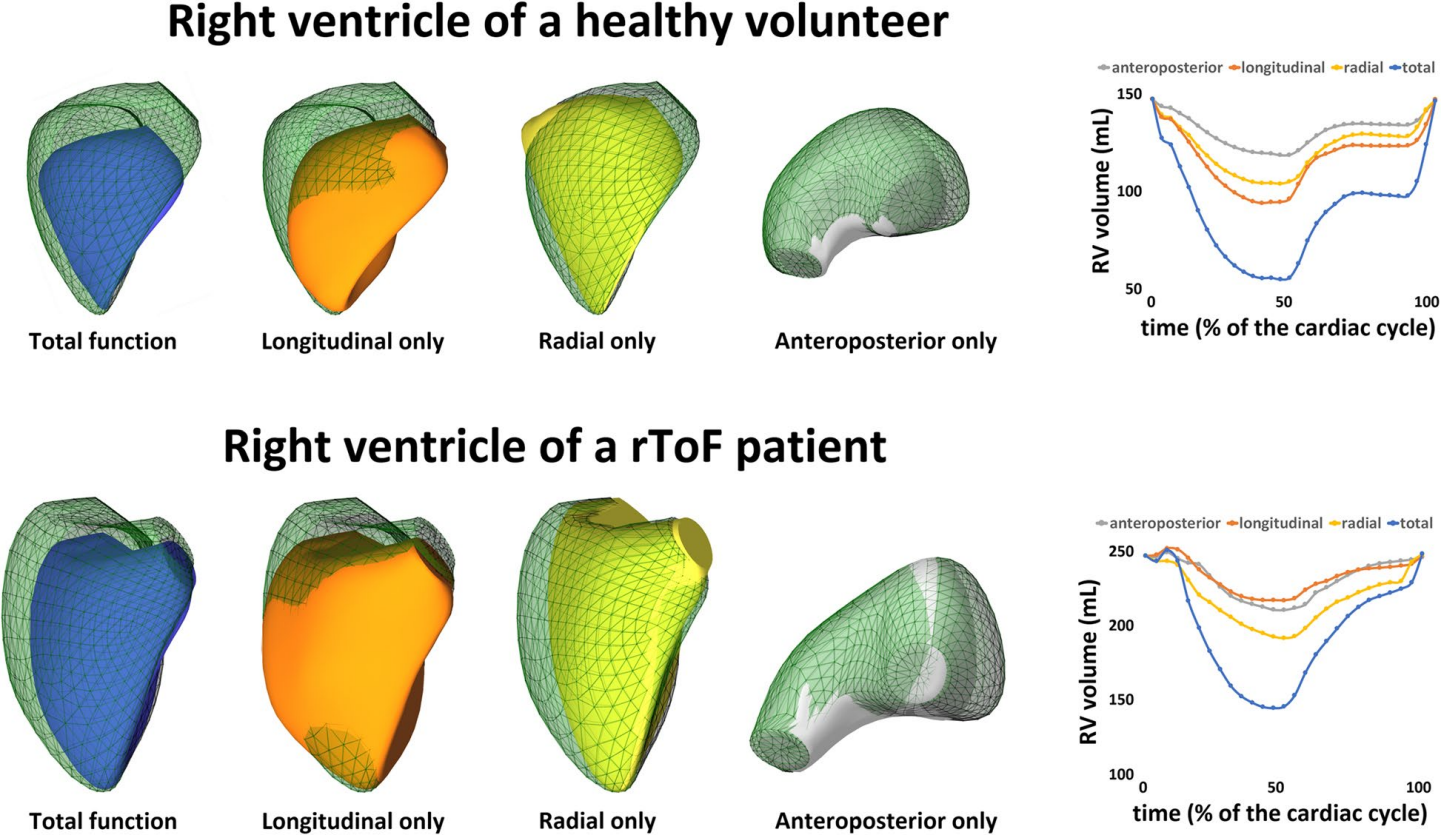
The motion of the right ventricular wall during the cardiac cycle in healthy volunteer and rToF patient can be decomposed in longitudinal, radial and anteroposterior axes, and the change of the volume during the cardiac cycle can be measured for each axis separately. The green mesh represents end-diastolic volume, and the blue surface is the end-systolic volume with all motion directions enabled. The orange surface represents the volume loss at end systole generated by only the longitudinal motion. The yellow surface represents the volume loss at end systole generated by only the radial motion and the grey surface—the volume loss at end systole generated by only the anteroposterior motion. One beat global (blue line) and decomposed volume-time curves of the right ventricle show that in healthy volunteers the longitudinal and radial motion contributes almost equally to global right ventricular function, while in rToF patients, the relative contribution of right ventricular longitudinal motion is impaired more than the radial or anteroposterior one. rToF – repaired tetralogy of Fallot

3D RV models can be exported from the commercially available TomTec software serving as input to assess the longitudinal, radial, and anteroposterior RV wall motions and their relative contribution to global RVEF using the ReVISION method (Right VentrIcular Separate wall motIon quantificatiON; Argus Cognitive, Inc., Lebanon, New Hampshire, USA; www.revisionmethod.com) [16] (Fig. 1). Briefly, the orientation of the exported 3D RV models is aligned using a standard, automated method to define the anatomically relevant, orthogonal axes (i.e., longitudinal, radial, anteroposterior). Then, the wall motions of the 3D model are splitted based on the movement of the model's each vertex point along these axes. Movement in each direction can be selectively switched on and off to assess the contribution of those which remained enabled. I.e., for quantifying the magnitude of the longitudinal motion, we take into account the movement of the vertices along the RV vertical (longitudinal) axis only. Thus, volume changes attributable to either longitudinal, radial, or anteroposterior directions can be separately quantified and the corresponding ejection fraction values can be calculated. Finally, the relative contribution of the longitudinal (LEF), radial (REF), or anteroposterior (AEF) RV wall motion to global RV pump function can be expressed by the ratio of the given directional ejection fraction to global RVEF. Global and regional 3D LS and circumferential (CS) strains were calculated as previously described [17].

### Statistical analysis

The qualitative variables were presented using absolute numbers and percentages. The continuous variables were summarized using mean value ± standard deviation. The RV strain parameters were considered negative (lower strain values indicate better deformation).

The distribution of variables was assessed by Kolmogorov–Smirnov test. Group comparisons (patients vs controls) were performed using Student’s t-test for normally distributed variables and Mann–Whitney U tests otherwise. Wilcoxon Signed Rank Test was used for the comparison of dependent variables. The correlations between RV mechanics and 2DTE, 3DTE and Doppler parameters of RV function (e.g. RV free wall S′, TAPSE, FAC) were assessed using Pearson or Spearman correlation coefficient, as appropriate (strain parameters were computed as absolute numbers).

To test the reproducibility in the computation of RV volumes, RV endocardial surface detection was repeated in 15 randomly selected rToF patients by the same observer and by a second independent observer, both blinded to all prior measurements. The inter- and intra-operator variability for RV volumes was computed using Lin’s concordance correlation coefficient.

Statistical analysis was performed using SPSS version 24.0 for Windows (SPSS, Chicago, IL, USA). Differences among variables and correlations were considered significant if p < 0.05.

## Results

Table 1 summarizes the demographic, clinical and echocardiographic characteristics of patients and controls. As expected, rToF and control groups were of the same age and gender. However, rToF patients had a smaller body surface area than healthy volunteers. Left ventricular ejection fraction was lower in rToF patients than in controls, but it was still within the normal range. rToF patients showed significantly larger RV volumes, lower RVEF, TAPSE, RV free wall S’ velocity, FAC and higher RV systolic pressure compared with the control group.

**Table 1:** Baseline clinical characteristics and right ventricular function parameters of patients with repaired tetralogy of Fallot and controls

Characteristics	rToF (n = 33)	Controls (n = 30)
Age (years)	20 ± 8	21 ± 6
Female, n (%)	19 (58)	19 (63)
Body surface area (m2)	1.6 ± 0.2	1.8 ± 0.3*
LV end-diastolic volume (ml/m^2^)	75 ± 13	65 ± 12**
LV end-systolic volume (ml/m^2^)	32 ± 7	25 ± 6***
LV ejection fraction (%)	58 ± 4	62 ± 4**
RV systolic pressure (mmHg)	32 ± 7	21 ± 5 **
RV free-wall S′ (cm/s)	10 ± 2	15 ± 2**
TAPSE (mm)	18 ± 3	25 ± 4**
RV fractional area change (%)	37 ± 5	49 ± 7**
RV end-diastolic volume (ml/m^2^)	133 ± 34	52 ± 18**
RV end-systolic volume (ml/m^2^)	74 ± 24	24 ± 10**
RV ejection fraction (%)	45 ± 6	55 ± 5**
Two-dimensional RV strain	**rToF (n = 29)**	**Controls (n = 28)**
RV global LS (%)	-20.0 ± 2.3	-24.9 ± 3**
RV free-wall LS (%)	-19.8 ± 3	-29.4 ± 3.9**
RV septal LS (%)	-18.7 ± 6	-19.7 ± 2.0
Age (months) at surgical repair	8 ± 11	-
Duration (years) from the surgery to the time of study recruitment	18.6 ± 7.3	-
Type of surgery		
Transannular patch	25 (76%)	-
Infudibulectomy	4 (12%)	-
Unknown	4 (12%)	-

*LS* longitudinal strain, *LV* left ventricular, *rToF* repaired tetralogy of Fallot, *RV* right ventricular, *TAPSE* tricuspid annular plane systolic excursion. *p < 0.05, **p < 0.01, ***p < 0.001

2D regional analysis of RV deformation was technically feasible in 29 rToF patients and 28 controls (Table 1). rToF patients demonstrated lower RV 2D global and RV free wall LS compared to controls, while the septal LS was similar in both groups.

### Three-dimensional right ventricular global and regional mechanics in rToF and controls

**Fig. 2:**
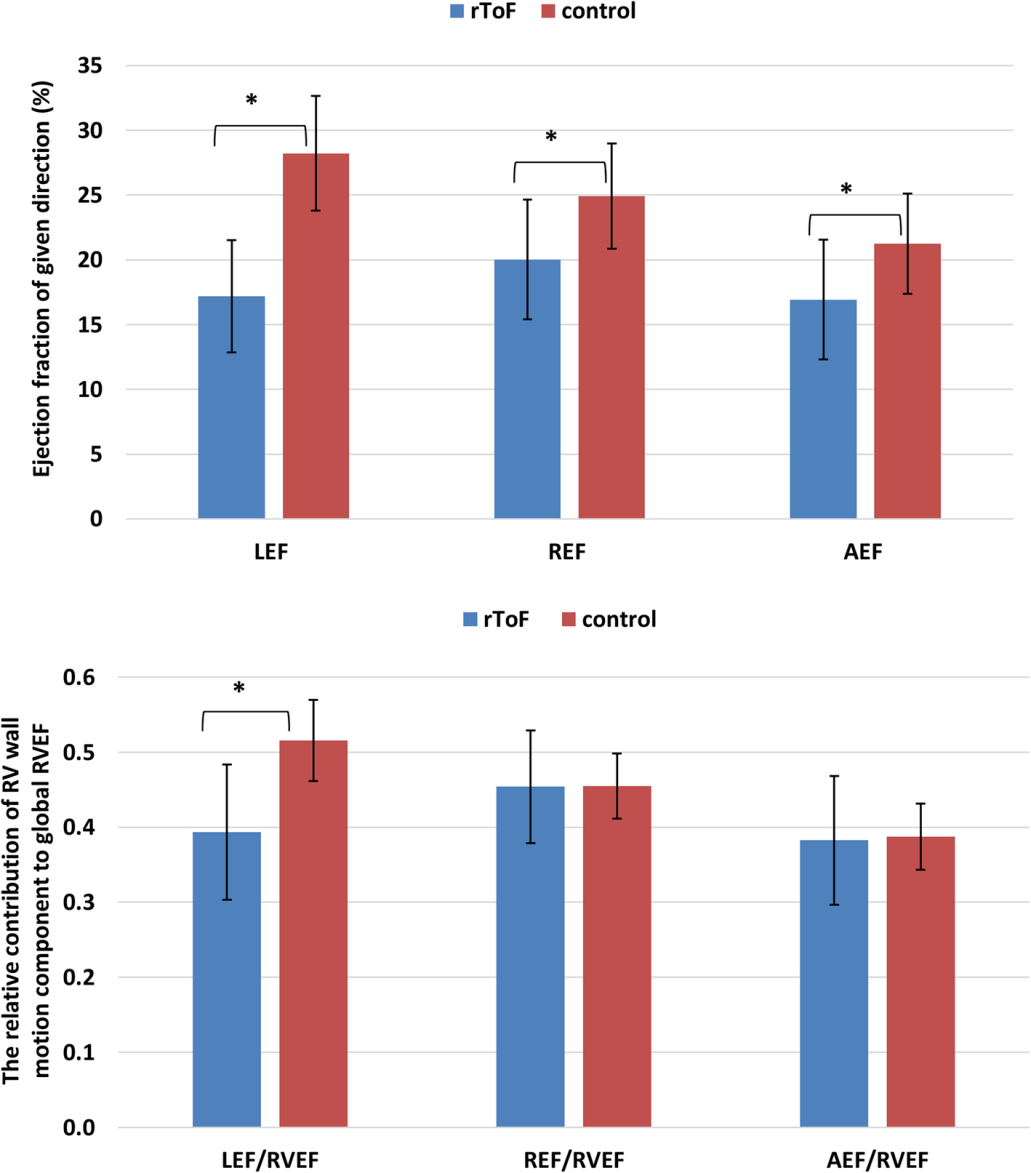
The right ventricular (RV) longitudinal ejection fraction (LEF), radial ejection fraction (REF) and anteroposterior ejection fraction (AEF), and their relative contribution to global right ventricular function (RVEF) (LEF/RVEF, REF/RVEF and AEF/RVEF, respectively) in the two study groups. rToF patients demonstrated impaired LEF, REF and AEF comparing with controls. However, in rToF the relative contribution of LEF to RVEF was significantly decreased, whereas the contribution of REF and AEF to RVEF was similar to controls. * p < 0.0001

3D echocardiographic parameters of RV mechanics revealed significant differences between the groups (Table 2, Figs. 1 and 2). Corresponding to decreased global RVEF, RV LEF, REF and AEF were significantly lower in rToF patients compared to controls. However, looking at the relative contribution of the different components of RV wall motions to global RVEF, rToF demonstrated significantly smaller relative LEF contribution to RVEF in comparison with the control group. Conversely, the relative REF and AEF contribution to global RVEF were similar between the groups.

**Table 2:** Three-dimensional global and regional right ventricular deformation data of patients with repaired tetralogy of Fallot and controls

Parameters	rToF (n = 33)	Controls (n = 30)
**RV LEF (%)**	17.2 ± 4	28.2 ± 4*
**RV REF (%)**	20.0 ± 5	24.9 ± 4*
**RV AEF (%)**	16.9 ± 5	21.2 ± 4*
**LEF/RVEF**	0.39 ± 0.09	0.52 ± 0.05*
**REF/RVEF**	0.45 ± 0.08	0.45 ± 0.04
**AEF/RVEF**	0.38 ± 0.09	0.39 ± 0.04
	**rToF (n = 32)**	**Controls (n = 29)**
**RV global CS (%)**	-19.8 ± 3.3	-22.8 ± 3.5*
**RV septal CS(%)**	-20.1 ± 5.7	-18.5 ± 3.6
**RV free-wall CS (%)**	-19.9 ± 3.5	-23.0 ± 3.5*
**RV free-wall apical CS (%)**	-20.0 ± 3.6	-22.1 ± 3.4
**RV free-wall mid CS (%)**	-20.3 ± 3.6	-22.5 ± 3.4
**RV free-wall basal CS (%)**	-19.9 ± 3.5	-23.0 ± 3.5*
**RV global LS (%)**	-16.9 ± 2.9	-23.2 ± 2.9**
**RV septal LS (%)**	-18.4 ± 3.7	-20.1 ± 3.8
**RV free-wall LS (%)**	-20.0 ± 3.4	-27.5 ± 4.0**
**RV free-wall apical LS (%)**	-20.4 ± 3.3	-26.5 ± 4.1**
**RV free-wall mid LS (%)**	-20.5 ± 3.4	-27.6 ± 4.3**
**RV free-wall basal LS (%)**	-20.0 ± 3.4	-27.5 ± 4.0**

*AEF* anteroposterior ejection fraction, *CS* circumferential strain, *LEF* longitudinal ejection fraction, *LS* longitudinal strain, *REF* radial ejection fraction, *rToF* repaired tetralogy of Fallot, *RV* right ventricular, *RVEF* right ventricular ejection fraction. *p < 0.01, **p < 0.0001

3D regional analysis of RV deformation parameters was technically feasible in 32 rToF patients and 29 controls (Table 2). Corresponding to decreased global RVEF, rToF patients demonstrated lower global 3D RV LS and CS compared with the control group. However, looking at the regional RV deformation, rToF patients had similar 3D RV CS values ​​in the septum, apical and middle RV free wall segments, and lower CS values ​​were found only in the basal RV free wall segment. 3D RV LS was reduced in all RV free wall segments in rToF, but it was similar to controls in the septal segments.

### The relationship between conventional echocardiographic parameters, global and regional RV mechanics and RV ejection fraction in rToF patients

In rToF patients, RV free wall S’ velocity (r = 0.356, p = 0.049), FAC (r = 0.556, p = 0.001), 2D RV global (r = 0.399, p = 0.032) and septal (r = 0.470, p = 0.01) LS demonstrated moderate correlation with global RVEF (strain parameters were computed as absolute numbers). TAPSE and 2D RV free wall LS did not show any significant correlation with RVEF (p > 0.05).

Conversely, global and regional RV mechanics assessed by 3DTE, demonstrated stronger correlations with global RVEF than the RV function parameters obtained by 2DTE (Table 3). In rToF patients REF and AEF showed better correlations with RVEF, than LEF. Whereas in the control group LEF (r = 0.748), REF (r = 0.834) and AEF (r = 0.748) showed quite similar strong positive correlation with global RVEF (p < 0.0001).

**Table 3:** Correlations between 2D echocardiography derived right ventricular function parameters and right ventricular global and regional mechanics assessed using 3D echocardiography in rToF patients

RV function parameters	RV longitudinal EF	RV radial EF	RV antero-posterior EF	3D RV global LS (%)	3D RV septal LS (%)	3D RV FW LS (%)	3D RV global CS (%)	3D RV septal CS (%)	3D RV FW CS (%)
TAPSE (mm)	r = 0.433*	r = 0.046	r = 0.085	r = 0.705*	r = 0.450*	r = 0.633*	r = 0.036	r = 0.173	r = 0.097
RV FW S′ (cm/s)	r = 0.415*	r = 0.416*	r = 0.050	r = 0.544*	r = 0.275	r = 0.484*	r = 0.104	r = 0.001	r = 0.001
RV FAC (%)	r = -0.173	r = 0.726*	r = 0.390*	r = 0.161	r = 0.374*	r = 0.158	r = 0.558*	r = 0.142	r = 0.531*
2D RV global LS (%)	r = 0.360	r = 0.109	r = 0.225	r = 0.448*	r = 0.305	r = 0.518*	r = 0.489*	r = 0.378*	r = 0.506*
2D RV FW LS (%)	r = 0.480*	r = 0.003	r = 0.015	r = 0.513*	r = 0.135	r = 0.483*	r = 0.262	r = 0.241	r = 0.267
2D RV septal LS (%)	r = 0.182	r = 0.269	r = 0.253	r = 0.192	r = 0.498*	r = 0.379*	r = 0.414*	r = 0.440*	r = 0.398*
3D RV EF (%)	r = 0.388*	r = 0.694*	r = 0.606*	r = 0.629*	r = 0.625*	r = 0.531*	r = 0.555*	r = 0.361*	r = 0.498*

*2D* two-dimensional, *3D* three-dimensional, *CS* circumferential strain, *EF* ejection fraction, *FAC* fractional area change, *FW* free wall, *LS* longitudinal strain, *rToF* repaired tetralogy of Fallot, *RV* right ventricular, *TAPSE* tricuspid annular plane systolic excursion. *p < 0.05

The relative LEF contribution to global RVEF had a moderate positive correlation with TAPSE (r = 0.359, p = 0.04) and negative correlation with FAC (r = -0.479, p = 0.005). The relative REF contribution to RVEF correlated moderately only with FAC (r = 0.509, p = 0.002), whereas the relative contribution of AEF to RVEF did not show a statistically significant correlation with traditional RV function parameters (p > 0.05).

3D RV global LS (r = 0.629 vs r = 0.399; p < 0.05), free wall LS (r = 0.531, p = 0.002 vs r = 0.264, p > 0.05) and septal LS (r = 0.625 vs r = 0.470, p < 0.05) showed stronger correlations (strain parameters were computed as absolute numbers) with RVEF than 2D LS. 3D RV global CS (r = 0.555, p < 0.001), free wall CS (r = 0.498, p = 0.004) and septal CS (r = 0.361, p = 0.042) showed moderate correlation with RVEF.

Among the 2DTE derived RV function parameters, FAC and RV free wall S’ velocity demonstrated moderate-strong positive correlation with RV REF (Table 3). RV 3D global (r = 0.521) and free wall CS (all segments r = 0.497; apical segment r = 0.504, middle segment r = 0.531 and basal segment r = 0.497) demonstrated a moderate correlation with REF (the deformation parameters were computed as absolute numbers) (p < 0.01). Only the septal 3D RV CS had no significant correlation with REF.

RV LEF showed moderate positive correlation with TAPSE, RV free wall S’ velocity and 2D RV free wall LS (Table 3). 2D RV global and septal LS did not show a significant correlation with LEF (p > 0.05). 3D RV global (r = 0.760) and free wall (all segments r = 0.652; apical segment r = 0.572, middle segment r = 0.597 and basal segment r = 0.652) LS demonstrated moderate-strong correlation with LEF (p < 0.01). 3D RV septal LS had no significant correlation with LEF (p > 0.05).

From 2DTE derived RV function parameters, only FAC had a weak positive correlation with AEF in rToF patients (Table 3). Meanwhile 3D RV global CS (r = 0.457), septal CS (r = 0.430) and free wall CS (all segments r = 0.475; apical r = 0.488, mid r = 0.477 and basal segment r = 0.475) showed a moderate correlation with AEF (p < 0.05).

### Reproducibility

Analysis of repeated measurements showed good reproducibility: the values for intra-operator and inter-operator variability of the 3D RV end-diastolic and end-systolic volumes were 0.944 (CI 0.867–0.977), 0.855 (CI 0.693–0.935) and 0.955 (CI 0.891–0.982) and 0.891 (CI 0.767–0.951), respectively. As the ReVISION method is a fully automated technique, it adds no further variability on top of the commercially available software for 3-D model reconstruction [18, 19].

## Discussion

Since conventional echocardiography explores mainly the longitudinal component of RV systolic function, there was no comprehensive method to evaluate the relative contribution of the different components of RV wall motion (e.g. longitudinal, radial and anteroposterior) to global RVEF. Thus, to our knowledge, this is the first study to assess 3DTE-derived global and regional RV mechanics in rToF patients with severe pulmonary regurgitation and their relations with RV pump function. According to our results: *i*) rToF patients had lower global RVEF compared to age- and sex-matched controls; *ii)* in terms of the relative contribution to global RVEF, only the longitudinal component of RV wall motion was reduced in rToF, whereas the radial and antero-posterior components were similar between rToF and controls; *iii*) rToF patients had similar RV septum, free-wall apical and mid segment 3D circumferential strain values, but significantly lower circumferential strain in the basal RV free wall segment*; iv)* RV 3D longitudinal strain values were reduced in the RV free wall segments in rToF, but they were similar to controls in the septum.

### RV function and mechanics in rToF patients

Recent studies demonstrated that longitudinal RV deformation is decreased in rToF with pulmonary regurgitation as a sequel [3, 20]. In addition, the deterioration of RV longitudinal strain has been found in rToF patients with maintained RVEF [3], suggesting that probably other wall motion components maintain the global RV function. However, despite the notable amount of circumferentially oriented myofibers in the subepicardial layer of the RV myocardium [21] and the complex RV contraction pattern[7], data about the non-longitudinal shortening of the chamber in rToF patients are scarce. Similar to the findings of Stephensen et al. [22], we found that in rToF patients with RV volume overload the longitudinal component of RV wall motion is impaired more than the radial one. According to our results, in comparison with the controls, the rToF patients had preserved the relative contribution of the radial (the so-called “bellows effect”) and anteroposterior motions to global RVEF, but significantly lower longitudinal shortening contribution to RVEF. Considering the decreased global RVEF in rTof patients, we can conclude that the radial and anteroposterior RV wall motion components have a higher impact to global RVEF in these patients. Thus, this could explain the phenomenon in clinical practice why some rToF patients with reduced TAPSE still have maintained global RVEF.

Furthermore, we demonstrated, that in rToF patients, the radial and anteroposterior components of ejection fraction showed better correlation with global 3D RVEF than the longitudinal one. These findings also emphasize that parameters measuring the longitudinal contraction offer only a partial assessment of the global RV pump function in rToF patients. This is very important because, due to the large surface of RV free wall, even a small amplitude of inward movement of the RV free wall may have a significant effect on the global RV function [4]. However, despite the evidences of the importance of the radial RV motion in physiological conditions and in patients with different cardiovascular diseases [4, 18, 23, 24], there is no 2D RV functional parameter, which allows to selectively and sensitively assess the relative importance of the radial contraction of the RV. Parameters used in everyday clinical practice refer predominantly to the longitudinal shortening of the RV wall (TAPSE, RV free-wall s′ velocity by tissue Doppler imaging). Moreover, the widely used TAPSE is a one-dimensional and angle-dependent parameter [25], which measures the excursion of the tricuspid annulus towards a reference external to the heart and ignores the outlet portion and the septal contribution to RV ejection. Selly et al. also confirmed that TAPSE is not sensitive enough to evaluate RV systolic function in rToF patients, whereas FAC, which incorporates longitudinal and (partly) radial components of RV contraction, and of course, 3D RVEF seems to correlate well with cardiac magnetic resonance imaging-derived measurements [26]. However, FAC still represents the RV function in only a single 2D cut plane exploring a very limited amount of the RV myocardium. Moreover, suboptimal RV endocardial definition, especially in dilated and heavily trabeculated RVs, can further reduce its accuracy. Of note, FAC does not include the contribution of the RV outflow tract to ejection, which is very important in patients with surgically repaired ToF, and can result in an overestimation of the global RV function in this subset of patients [5].

Speckle-tracking echocardiography has been shown to be more sensitive than conventional measures in detecting changes in myocardial function in rToF [27]. However, although 2D longitudinal strain is less load-dependent than conventional measures of RV function, it still explores the deformation of the myocardium in one direction only. In rToF patients, RV 2D longitudinal strain (measured from the apical four-chamber view) showed only a weak correlation with RVEF [28]. Accordingly, in our rToF patients, RV 2D global and septal LS had a weak to moderate correlation with RVEF, but RV free wall LS did not show a significant correlation with RVEF. This is probably related to measuring only the inlet and trabecular parts of the RV, and also the fact that radial/anteroposterior RV shortening is not taken into account. Estimating the RV circumferential strain in 2D requires a short axis view, which is difficult to obtain in adults. 3DTE allows us to encompass the entire RV in a single pyramidal dataset and perform a detailed quantitative analysis of its size and function providing a good correlation with RV volumes and ejection fraction measured by cardiac magnetic resonance [8]. Compared with 2D strains, 3D analysis is not slice-plane limited, delivers vectored data in 3 orthogonal planes from one single dataset and given the complex anatomy of the RV, may better reflect the true pattern of RV contraction. Recently, Moceri et al. [29] demonstrated that rToF patients with chronic RV volume overload had lower RV 3D global longitudinal (the inlet septum and superior part of the free wall were preserved) and circumferential (the infundibular, membranous and inlet septum were preserved) strain compared with controls. Accordingly, we also found that in rToF patients with severe pulmonary regurgitation RV 3D global longitudinal and circumferential (summation of radial and anteroposterior motions) strain were reduced compared with controls. However, according to our data, looking at the regional RV deformation, rToF patients had similar RV septum, free wall apical and mid segment CS values, and lower CS only in the basal RV free-wall segment. We hypothesize that these findings may explain the preserved RVEF in the apical region demonstrated by Van Der Hulst et al [30]. Moreover, we found that RV LS was reduced in the RV free wall segments in rToF, but it was similar to controls in the septum. Finally, we showed that 3D LS had better correlation with the LEF and global RVEF compared with 2D strain, in rToF patients.

The RV anteroposterior shortening is even more neglected in daily clinical practice, thus, data about this component of global RVEF are highly limited. We hypothesize that RV anteroposterior shortening may reflect the left ventricular contraction’s contribution to overall RV function by stretching the RV free wall insertion lines over the interventricular septum. Recently, Lakatos et al. demonstrated comparable RV AEF values between heart transplant recipients and healthy control subjects, and also between elite athletes and sedentary individuals [18, 19]. According to our results, the relative contribution of AEF to global RVEF in rToF was similar to controls. The common feature of both rToF and controls is that the left ventricular function was preserved, which could be related to the maintained anteroposterior RV shortening.

There are several underlying mechanisms which may explain the RV mechanical remodeling we observed in rToF patients with severe pulmonary regurgitation. First, according to the Frank-Starling law, when preload increases and the RV dilates, the contractile function increases and, for some time, effectively compensates the altered hemodynamic conditions. However, long-term chronic RV volume overload leads to stretching of myofibers with subsequent distorted meshwork, more spherical architecture and re-alignment of myocardial fibers that might modify the contraction pattern to a greater radial contribution [22, 31]. Previous studies also suggest another possible explanation for mechanical remodeling in rToF patients. Compared to healthy individuals, ToF patients have a prominent middle layer with circumferentially oriented myofibers, which resembles the left ventricular architecture [32]. Moreover, it was shown that global and regional RV myocardial deformation was affected differently by chronic volume loading in patients with atrial septal defect compared to ToF patients [29, 33]. Data exist on the improvement of longitudinal strain after percutaneous pulmonary valve replacement in ToF patients [27, 34]. Thus, future studies are needed to determine whether these changes in myofiber architecture and subsequently in the mechanical pattern are inherited or just consequences of chronic volume overload of the chamber.

### Limitations

The application of the ReVISION method relies on accurately generated 3D endocardial surface model and therefore, it is dependent on the quality of the 3DTE images. Moreover, in rToF, the acquisition of good 3D datasets could be hampered by a markedly dilated RV. However, experience in 3D acquisition and analysis might minimize this limitation. Nevertheless, ReVISION method assess RV mechanics in a fully automated manner and does not add further variability to that associated with 3D RV volumetric and functional evaluation.

Strain measurements based on 3D analysis cannot be compared with measurements performed using commercially available 2D methods. Moreover, the absence of a true reference method to test the accuracy of our measurements could be considered as a limitation of this study, and only prospective outcome studies can assess the clinical value of our approach.

## Conclusions

According to our knowledge, this is the first study to evaluate the relative changes in RV mechanics in rToF patients, and their relations with global RV pump function using 3DTE.

We found that, in rToF patients with RV volume overload due to severe pulmonary regurgitation, the longitudinal component of RV wall displacement is affected more than the radial or anteroposterior one. Moreover, among the longitudinal components of RV function, only the longitudinal function of the RV free wall seems to be affected, whereas the interventricular septum is spared. Finally, global RVEF shows weak correlations with parameters that reflect mainly longitudinal RV shortening. Thus, our results emphasize that, conventional echocardiographic parameters, usually describing only the longitudinal function of the RV, might be insufficient to accurately assess the RV pump function in rToF patients. Conversely, 3D RVEF incorporates the contribution of all motion directions (longitudinal, radial and anteroposterior), thus, 3DTE may be the echocardiographic method of choice to accurately measure global RV function in rToF patients.

Moreover, 3DTE may provide additional pieces of information which are not available using conventional parameters and improve our understanding of the mechanisms leading to RV failure in rToF patients. However, larger future studies are needed to confirm these findings, and to determine the potential predictive value of the different components of global RVEF on the outcome of rToF patients.

## Data Availability

The datasets used and/or analysed during the current study are available from the corresponding author on reasonable request.

## Abbreviations

2DTE: Two-dimensional transthoracic echocardiography
3DTE: Three-dimensional transthoracic echocardiography
AEF: Anteroposterior ejection fraction
CS: Circumferential strain
FAC: Fractional area change
LEF: Longitudinal ejection fraction
LS: Longitudinal strain
REF: Radial ejection fraction
RV: Right ventricle/ventricular
RVEF: Right ventricular ejection fraction
rToF: Repaired tetralogy of Fallot
TAPSE: Tricuspid annular plane systolic excursion
